# Internal Teat Sealant Administered at Drying off Reduces Intramammary Infections during the Dry and Early Lactation Periods of Dairy Cows

**DOI:** 10.3390/ani10091522

**Published:** 2020-08-28

**Authors:** Gustavo Freu, Tiago Tomazi, Camylla Pedrosa Monteiro, Melina Melo Barcelos, Bruna Gomes Alves, Marcos Veiga dos Santos

**Affiliations:** 1 Department of Animal Nutrition and Production, School of Veterinary Medicine and Animal Science, University of São Paulo (USP), Pirassununga, São Paulo 13635-900, Brazil; gustavofreu@usp.br (G.F.); tt489@cornell.edu (T.T.); camyllapedrosa@hotmail.com (C.P.M.); melina.barcelos@usp.br (M.M.B.); bgalves@usp.br (B.G.A.); 2 College of Veterinary Medicine, Federal Institute of Santa Catarina, Concórdia, Santa Catarina 89703-720, Brazil; 3 Department of Population Medicine and Diagnostic Sciences, College of Veterinary Medicine, Cornell University, Ithaca, NY 14853, USA

**Keywords:** bovine mastitis, clinical mastitis, bacteriological cure, new intramammary infections, dry period, bismuth subnitrate

## Abstract

**Simple Summary:**

Internal teat sealant (ITS) at drying off is a strategy used for the prevention of intramammary infections (IMI) during the dry period (DP), as it simulates the keratin plug’s purpose, which is to prevent the access of pathogens into the mammary gland. The results from randomized clinical trials provide valuable information about the efficacy of commercially available products to be used in dairy cattle, assisting farmers to choose the best protocols for mastitis control. This study evaluated a new ITS infused at drying off as an alternative to prevent IMI during DP in a tropical country. Our results showed no effect of treatments on risk of bacteriological cure, subclinical mastitis (SCM) cure, and new cases of SCM postpartum. On the other hand, the use of ITS combined with an intramammary antibiotic (SDCT) reduced the risk of clinical mastitis up to 60 days postpartum, the overall risk of new intramammary infections (NIMI), and the NIMI caused by major pathogens compared to the use of antimicrobial alone (ADCT). Thus, the use of ITS combined with an antimicrobial at drying off was effective to prevent NIMI and clinical mastitis up to 60 days postpartum.

**Abstract:**

The effect of an internal teat sealant (ITS) on subsequent infection of the mammary gland was evaluated on the following mammary gland health indicators: (a) bacteriological cure of preexisting intramammary infections at drying off, (b) risk of postpartum new intramammary infections (NIMI), (c) cure and risk of new cases of subclinical mastitis (SCM), and (d) risk of postpartum clinical mastitis (CM). A total of 553 cows during late gestation were randomly assigned into two treatment protocols at drying off: (a) Dry cow therapy with 0.25 g of intramammary anhydrous cefalonium (ADCT; Cepravin^®^, MSD Animal Health); or (b) ADCT combined with ITS (SDCT; 4 g bismuth subnitrate; Masti-Seal^®^, MSD Animal Health, São Paulo, Brazil). Mammary quarter (MQ) milk samples were collected for microbiological culture and somatic cell count (SCC) at drying off and early lactation, and data from 1756 MQ were used in the multivariate logistic regression. There was no effect on the risk of bacteriological cure, SCM cure, and new cases of postpartum SCM. Still, SDCT reduced the risk of CM up to 60 days postpartum (DPP), overall NIMI risk, and the NIMI caused by major pathogens compared to ADCT. Thus, the DCT combined with ITS at drying off is effective for preventing NIMI during the dry period and CM up to 60 DPP.

## 1. Introduction

During the dry period of dairy cows, the mammary gland (MG) undergoes physiological and histological changes that affect udder health and milk production of the next lactation [1,2,3]. The dry period is also a good opportunity to treat intramammary infections (IMI) acquired during lactation, although it is also a phase of high risk for the development of new intramammary infections (NIMI; [4]) that can evolve to clinical mastitis (CM) in subsequent lactation [5].

The risk of NIMI during the dry period is increased when there is a non- or malformation of the keratin plug in the teat canals, which acts as a physical barrier against microorganisms [6,7]. However, the production of this keratin plug varies between cows [8]; 23.4% of the mammary quarter (MQ) remain open until six weeks after drying off [9], whereas 5% have no plug during the whole dry period [10]. Mammary quarters without keratin plug formation have 1.7 times greater chance of NIMI during the dry period than those with complete formation of this natural barrier [9]. In addition, MQs infected during the dry period have a higher risk of postpartum CM [11], and lower milk yield compared to uninfected MQs during the dry period [12].

The use of internal teat sealant (ITS) at drying off is a strategy for IMI prevention during the dry period, as it simulates the keratin plug and reduces microorganisms’ access to the MG [13]. The combination of ITS with dry cow therapy (DCT) at drying off has also been shown effective in the control of NIMIs, compared to untreated MQ [13] or those receiving only antibiotics [14]. Studies with ITS have been reported in Europe [15], Australia [14], and North America [16]. In a meta-analysis, Rabiee and Lean [17] reported that the infusion of ITS alone or combined with DCT reduced by 25% the NIMI risk in dairy cows after calving compared with those receiving only antibiotics. In addition, there was a 29% reduction in the risk of CM after calving when ITS was used alone or after DCT infusion compared with cows treated with antibiotics alone [17].

Although the use of ITS during the dry period is well studied in some countries, there is a growing demand for new products that can improve MG health. Randomized clinical trials evaluating new ITSs commercially available may provide information about the efficacy of specific formulations, which could help farmers make informed science-based decisions when selecting ITS products for use in their herds. In this context, the present study evaluated a new ITS as an alternative strategy to prevent NIMI during the dry period under tropical environmental conditions of milk production. The high temperatures and abundant rainfall in tropical countries are factors associated with higher bacterial counts in the environment [18]. In addition, the aforementioned factors can increase the risk of heat stress, which is associated with the impairment of cows’ immune defenses. Therefore, the use of ITS can be a suitable strategy to reduce the access of opportunistic microorganisms to the mammary gland via the teat canal. Our objective was to evaluate the efficacy of ITS formulated with bismuth subnitrate (Masti-Seal^®^; MSD Animal Health, São Paulo, Brazil) used in combination with an antibiotic (0.25 g of anhydrous cefalonium), administered at quarter level to dairy cows at drying off, on the risk of bacteriological cure, subclinical mastitis (SCM) cure, new SCM cases, NIMI, and CM until 60 days postpartum (DPP).

## 2. Materials and Methods

All experimental procedures using animals were carried out under approval of the Ethics Committee from the School of Veterinary Medicine and Animal Science at the University of São Paulo, Brazil (Protocol number: 7116230919).

### 2.1. Herd Selection and Cow Enrollment

The study was conducted from June 2017 to December 2018 in five dairy herds located in the Brazilian states of Minas Gerais (*n* = 3), São Paulo (*n* = 1), and Paraná (*n* = 1). At the beginning of the study, herds had an average of 570 lactating cows (ranging from 230 to 730) and milk production of 27.5 ± 3 L (mean ± SD)/cow/day. During lactation, cows were housed in free stall (*n* = 4) or compost bedded pack barn (*n* = 1) systems, while during the dry period, all cows were kept in open paddocks, received water ad libitum, and were fed according to the nutritional management of each herd.

The selected cows in this study were at late gestation (±220 days), with good clinical condition (i.e., no symptoms of disease), and did not receive systemic or intramammary antibiotic treatment or anti-inflammatory drugs 30 d before drying off. Cows should have an expected dry period length of 50 to 100 d. Before drying off, information such as herd of origin, breed, age, parity, days in milk (DIM), CM history, predicted calving date, and somatic cell count (SCC) history (last three months) was recorded for all enrolled cows. Cow-level analyses of SCC (i.e., composite milk samples) were carried out on each farm on a monthly basis and recorded in electronic spreadsheets. In addition, SCC history on each farm was recorded in the week before the inclusion of cows in the study.

### 2.2. Sample Size Determination

Sample size was determined based on a 25% postpartum NIMI risk reported at the quarter level in cows receiving only antibiotics at the dry off [19]. A total of 1514 mammary quarters were required to have an 80% chance of detecting, as significant at the 5% level, a decrease of 20% in the risk of NIMI between cows assigned to the control (only antibiotic) and experimental (antibiotic combined with ITS) dry-off protocols.

### 2.3. Treatment Allocation and Administration

The selected cows were randomly assigned to one of two treatment protocols according to a previously prepared randomized spreadsheet using the RAND function of Excel software (2010, Microsoft Office Corporation, Redmond, WA, USA). The selected cows were randomly assigned to receive one of the following treatments: (ADCT, antibiotic DCT) infusion of 0.25 g anhydrous cefalonium, (Cepravin^®^, MSD Animal Health, São Paulo, Brazil) in each mammary quarter; or (SDCT) infusion of the aforementioned antimicrobial followed by the infusion of ITS (4 g bismuth subnitrate, Masti-Seal^®^, MSD Animal Health, São Paulo, Brazil). For each farm, a form was created with the list of randomized order of treatment protocols, which was placed in the milking parlor or any other facility where the treatments were performed.

Prior to treatment administration, cows were completely milked and the teat ends were disinfected with 70% iodized alcohol. For ADCT, the intramammary antibiotic was infused into each teat followed by a massage in the vertical direction, and teats were dipped in a commercial iodine-based teat dip (1% iodine, Della Barrier, DeLaval, Tumba, Sweden). The same procedures were used in all farms. For SDCT, after antibiotic infusion, the ITS was administered in each teat. During the infusion of the ITS, the teat base was grasped with two fingers to assure that the ITS remained in the teat canal, and no massage was performed. After both treatments, milking was stopped abruptly. In all farms, trained farm personnel administered the treatments.

### 2.4. Milk Sample Collection

Mammary quarter milk samples were collected aseptically for bacteriology on the drying off day and at 7 ± 3 and 14 ± 3 DPP. Prior to milk collection, the teat end was cleaned and disinfected with 70% iodized alcohol, according to NMC [20] recommendations. The first milk strips were discarded and the milk was collected directly in a sterile tube, previously identified with collection date, farm name, cow number, and MQ. After milk collection, the samples were transported in an isothermal box (≃4 °C) to the laboratory and then, frozen (−20 °C) until microbiological analysis.

Samples for SCC were collected on the drying off day and at 14 ± 3 DPP in a 50-mL plastic tube containing 2-bromo-2-nitropropane-1,3-diol chemical preservative (Bronopol, Microtabs II, D&F Control Systems Inc., Norwood, MA, USA). After collection, the samples were homogenized and analyzed by flow cytometry using the Somacount 300^®^ equipment (Bentley Instruments Inc., Chaska, MN, USA).

Clinical mastitis cases were identified and recorded by trained farm personnel during the dry period up to 60 DIM. Cows with visible changes in the appearance of milk (presence of lumps, pus, clots, blood, or aqueous milk) and/or in the udder (edema, redness) were identified and recorded on forms containing cow identification, date of clinical case, and affected MQ.

### 2.5. Microbiological Analysis

Bacteriological analysis was performed according to procedures recommended by the NMC [20]. An aliquot of 0.01 mL of milk was streaked onto a blood agar plate containing 5% bovine blood. The plates were incubated at 37 °C for 48 h and morphological characteristics of colonies were observed every 24 h. Samples with growth of >2 types of microorganisms were considered as contaminated [4].

Catalase, KOH, and Gram staining tests were performed for differentiation of isolates. Isolates identified as catalase-positive and Gram-positive cocci were submitted for coagulase tests (Coagu-Plama, Laborclin, Brazil). Isolates with negative coagulase tests were defined as coagulase-negative staphylococci (CNS), while isolates with a positive reaction associated with a hemolytic characteristic in blood agar medium and latex agglutination were defined as *Staphylococcus aureus*.

For isolates identified as Gram-positive catalase negative cocci, tests of Christie–Atkins–Munch-Petersen (CAMP), esculin, growth in bile esculin, and pyrrolidonyl arylamidase (PYR) were performed to differentiate between *Streptococcus agalactiae*, *Streptococcus dysgalactiae*, *Streptococcus uberis*, other *Streptococcus*, and *Enterococcus* spp.

Gram-negative bacteria (e.g., *Escherichia coli*, *Klebsiella* spp., and *Pseudomonas* spp.) were identified using MacConkey agar (KASVI, São José dos Pinhais, Brazil) and confirmed with additional tests of oxidase, motility and gas, indole, Voges–Proskauer, urease, and ornithine decarboxylation. Oxidase-positive isolates were considered *Pseudomonas* spp., while oxidase-negative isolates were evaluated by a modified Rugai test (Enterex^®^, Cefar Diagnostic LTDA, São Paulo, Brazil) for presumptive identification of *Escherichia coli*, *Enterobacter* spp., *Serratia* spp., and *Klebsiella* spp. Colonies with rod morphology and pink staining on MacConkey agar were considered Gram-negative rods. Gram-positive rods were classified according to their micromorphology into *Corynebacterium* spp., *Nocardia* spp., *Trueperella pyogenes*, and *Bacillus* spp. Yeasts were identified by morphological characteristics with an optical microscopy [4].

### 2.6. Definition of Mammary Gland Health Indicators

Bacteriological cure of IMI: A MQ was considered bacteriologically cured when the microorganism identified at drying off was not isolated in either of the two postpartum milk samples (7 ± 3 and 14 ± 3 DPP).

Subclinical mastitis cure: This was considered when MQs with SCC >200,000 cells/mL before drying off had reduction to values ≤200,000 cells/mL after calving.

New SCM cases: These were defined when MQ had SCC ≤200,000 cells/mL before drying off, with an increase to >200,000 cells/mL after calving.

New intramammary infection: A NIMI was defined when a MQ with milk samples collected at drying off had negative culture (no growth), but a microorganism was identified in either of the two milk samples collected postpartum; or if a different pathogen than that isolated at drying off was recovered in either of the postpartum samples.

Clinical mastitis: This was defined by visual changes in milk accompanied or not by other clinical signs, such as udder swelling, redness, heat, and pain [21], identified by farmers of selected herds.

The following classification was performed based on the bacteriological group of postpartum NIMI-causing pathogens: (a) major pathogens (*Strep. uberis*, *Staph. aureus*, other Strep., *Strep. dysgalactiae*, *Strep. agalactiae*, *Escherichia coli*, *Klebsiella* spp., and *Pseudomonas* spp.); (b) minor pathogens (CNS and *Corynebacterium* spp.); (c) other microorganisms (other Gram-positive and Gram-negative bacteria, *Enterobacter* spp., yeast, *Enterococcus* spp., and *Bacillus* spp.).

When a milk sample had two different microorganisms isolated in the microbiological culture (i.e., mixed culture), the following interpretation was performed: isolation of major and minor pathogens was considered NIMI caused by a major pathogen; isolation of *Staph. aureus* and *Streptococcus* spp. was considered NIMI caused by *Staph. aureus*; isolation of *Corynebacterium* spp. and CNS was considered NIMI caused by CNS [22].

### 2.7. Statistical Analysis

Data analysis was performed using statistical software SAS version 9.4 (SAS Institute, Cary, NC, USA). Descriptive analyses were performed using PROC FREQ. Characteristics of cows and quarters assigned to the treatment groups were compared considering the day of cows’ enrollment in univariate analysis using the PROC FREQ; chi-squared test was used for categorical variables (i.e., quarter position) and PROC TTEST was used for continuous variables (i.e., parity, DIM, and quarter-level linear score of SCC; LSSCC [23]). The SCC data were transformed into LSSCC [24] to approximate the data to normal distribution using the Excel program (Microsoft Office, 2016):

$$\mathrm{LSSCC}={\mathrm{Log}}_{2}\left(\mathrm{SCC}/100\right)+3,$$

To evaluate the effect of treatment on the dichotomized outcomes variables, such as risk of bacteriological cure, SCM cure, new SCM cases, and NIMI (overall and by group of pathogens), logistic regression models were fitted with a binary distribution using PROC GLIMMIX of SAS (SAS Institute Inc., Cary, NC, USA). Herd and cow were included in the model as random effects. Independent variables (parity, DIM, dry period, and LSSCC at drying off) were tested using univariate analyses, and only those with *p* ≤ 0.30 were maintained in a multivariable model [4]. The final model was obtained after performing a manual selection and elimination procedure.

Potential confounders were monitored by changes in the coefficient of DCT after removing another variable from the model, as well as the adjustment parameters (AIC and −2 Log L). When a change over 25% was observed in the treatment coefficients for cure risk (bacteriological and SCM), new cases of SCM and NIMI overall and by pathogens group, the variable was reinserted into the model. Effect of treatments on response variables was assessed at the MQ level using the following binary logistic regression model:

$$\begin{array}{cc} \mathrm{logit}\;\left(\mathrm{pi}\right)=\beta_{0}+ & \beta_{1}\times \mathrm{Treat}+\beta_{2}\times \mathrm{Pos}+\beta_{3}\times \mathrm{Parity}\left(\mathrm{covariate}\right)+\beta_{4}\times \mathrm{DIM}\left(\mathrm{covariate}\right)\\ & +\beta_{5}\times \mathrm{DP}\left(\mathrm{covariate}\right)+\beta_{6}\times \mathrm{LSSCC}\left(\mathrm{covariate}\right)+\mathrm{cow}\left(\mathrm{random}\right)\\ & +\mathrm{herd}\left(\mathrm{random}\right)+e, \end{array}$$

where logit (pi) is the logistic function of cure risk (bacteriological and SCM), new infection or new SCM cases; β_0_ is the intercept; β_1_ = regression coefficient for the DCT protocol (ADCT or SDCT); Treat = treatments; β_2_ = regression coefficient for quarter position (front or rear); Pos = mammary quarter position; β_3_ = regression coefficient for parity as a covariate; β_4_ = regression coefficient for DIM included as a covariate; DIM = days in milk; β_5_ = regression coefficient for dry period duration included as a covariate; DP = dry period; β_6_ = regression coefficient for SCC at drying off transformed on linear logarithmic scale (LSSCC; included as covariate); Cow = random cow effect; Herd = random herd effect; e = residual error. Variables were considered statistically significant when a *p*-value ≤0.05 was detected. Data are reported as LSM ± SEM unless otherwise stated.

The effect of drying off protocols on the CM risk within the first 60 DPP was assessed using Cox regression for competing-risks data with cause-specific hazard of treatment (PHREG procedure, SAS version 9.4; [4]). The end of days at risk was defined as the day after calving that the MQ was identified with CM. The independent variables offered to the model were treatment, position of affected quarter, parity, and the interaction of treatment and parity. Herd was offered to the model as a random effect. A backward stepwise elimination procedure was performed and only variables with *p* < 0.10 were kept in the final model. Cows that presented any concomitant disease or that died before 60 DPP were not accounted for in this analysis.

## 3. Results

A total of 553 cows met the selection criteria and were included in this study (ADCT = 270 cows; SDCT = 283 cows). However, 114 cows (ADCT = 60; SDCT = 54) were excluded post admission and their data were not evaluated in the statistical analyses. There was no difference (*p* = 0.94) between groups on the number of cows excluded post admission. Reasons for a cow’s exclusion were: (a) involuntary culling (*n* = 57), (b) other diseases (e.g., metritis, placental retention, pneumonia, and lameness) requiring therapeutic interventions (*n* = 48), and (c) abortion (*n* = 9). A total of 439 cows (1756 MQ) had data evaluated. Of these, 210 (840 MQ) were assigned to ADCT and 229 (916 MQ) to SDCT.

**Figure 1:**
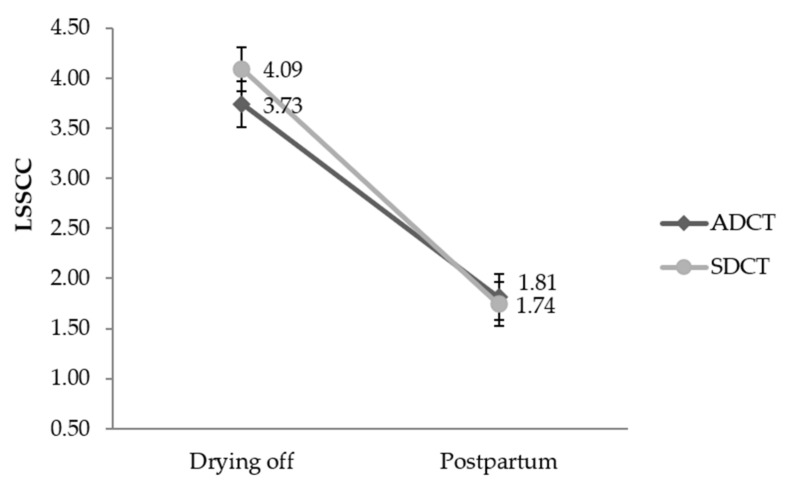
Descriptive results of mammary quarters SCC of samples collected at drying off and postpartum (mean and standard deviation) according to the treatment groups evaluated. *p*-value: Treatment: *p* = 0.292; Time: *p* < 0.001; Treatment vs. Time: *p* = 0.005; Days in milk: *p* = 0.0127; Parity: *p* < 0.0001. LSSCC: SCC transformed on linear score according to Schukken et al. [24]. ADCT: 0.25 g anhydrous cefalonium. SDCT: 0.25 g anhydrous cefalonium used in combination with ITS (bismuth subnitrate 4 g).

Of the total cows evaluated, 420 (95.7%) were Holstein, 13 (3.0%) Girolando crossbred (*Bos taurus* × *Bos taurus indicus*), and 6 (1.3%) Jersey. There was no statistical difference (*p* > 0.05) between treatment groups in the distribution of cows according to parity, dry period length, and MQ position (Table 1). On the other hand, cows from the ADCT group had higher DIM (*p* = 0.0007) and lower LSSCC (*p* = 0.001) compared to the SDCT group. There was a time effect (*p* < 0.001) and time vs. treatment interaction (*p* = 0.005) effect when comparing the SCC recorded at drying off with that assessed at postpartum (Figure 1).

### 3.1. Prevalence of Mastitis Pathogens Isolated at Drying off and Postpartum

In total, 1375 (78.30%) of 1756 MQs (439 cows) had negative culture results (i.e., no microbiological growth) at drying off. Among the positive cultures, CNS were the most frequent pathogens (*n* = 180; 10.25% of the total samples), followed by *Staph. aureus* (*n* = 51; 2.90%) and *Corynebacterium* spp. (*n* = 30; 1.71%). Only 21 samples had isolation of Gram-negative bacteria (Table 2).

After calving, a total of 1551 (88%; ADCT: 47%, SDCT: 53%) and 1547 (88%; ADCT: 46.15%, SDCT: 53.85%) MQs had negative culture at the first and second samples collected postpartum, respectively (Table 3). CNS, *Staph. aureus*, *Strep. uberis*, and *Streptococcus* spp. were the most frequently Gram-positive pathogens recovered from postpartum cultures. Among Gram-negative bacteria, the most prevalent were *Pseudomonas* spp., *Enterobacter* spp., and *Escherichia coli*.

### 3.2. Bacteriological Cure

**Figure 2:**
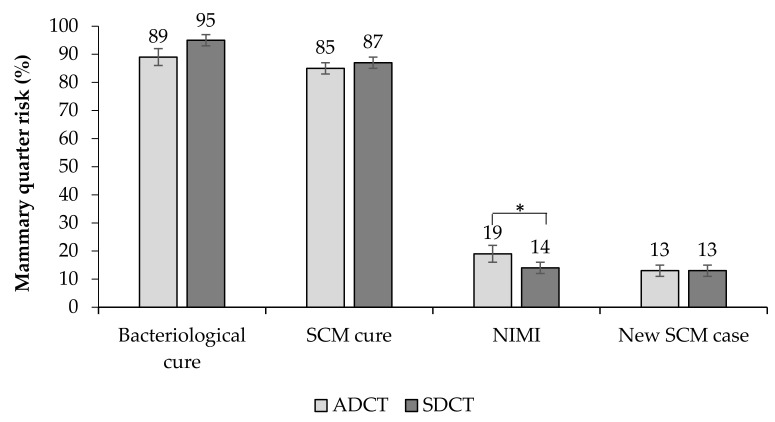
Risk of cure (bacteriological and of SCM), new SCM cases, and new intramammary infections (NIMI) according to the microbiological and somatic cell count (SCC) results of mammary quarters of 439 cows submitted to drying off protocols. *p*-value: Bacteriological cure: *p* = 0.09; SCM cure: *p* = 0.40; New intramammary infection: *p* = 0.007; New SCM cases: *p* = 0.93. * Variables followed by asterisks were considered significantly different (*p* < 0.05). ADCT: 0.25 g anhydrous cefalonium. SDCT: 0.25 g anhydrous cefalonium used in combination with ITS (bismuth subnitrate 4 g).

A total of 355 MQs were evaluated for bacteriological cure (ADCT = 171, SDCT = 184). The average risk of bacteriological cure was 91.3% (324/355 MQ) and no effect of ITS (*p* = 0.07; Table 4) was observed for this outcome. Cows assigned to the ADCT protocol had a bacteriological cure of 89.0%, while the SDCT group had 95.0% of bacteriological cure (Figure 2).

Bacteriological cure risk for Gram-positive pathogens was 91% (303/334 MQs), while for Gram-negative pathogens, it was 100% (21/21; Table 5). On the other hand, cows with isolation of CNS had the lowest bacteriological cure among pathogens isolated before treatment (ADCT = 76% and SDCT = 89%).

### 3.3. SCM Cure and New SCM

A total of 882 MQs were evaluated for SCM cure risk (ADCT = 378, SDCT = 504). The use of ITS did not affect (*p* = 0.40) the SCM cure risk, which had an overall average of 85.6% (755/882 MQs; Figure 2, Table 4). Similarly, there was no effect of treatments (*p* = 0.62) on the SCC postpartum.

Additionally, a total of 764 MQs were assessed for the risk of new SCM cases postpartum (ADCT = 399; SDCT = 365). Overall, the risk of new SCM cases was 13.3% (102/764 MQs), and no significant difference (*p* = 0.93) was observed between the ADCT (12%) and SDCT groups (13%; Figure 2, Table 4).

### 3.4. New Intramammary Infection

A total of 1711 MQs were assessed for NIMI risk (ADCT = 824, SDCT = 887). The NIMI risk was lower in SDCT-treated cows than ADCT-treated cows (14% vs. 19%, respectively; *p* = 0.007). According to the regression results, mammary quarters assigned to the ADCT protocol were 1.4 times more likely to develop NIMI than those treated with SDCT (Figure 2; Table 6). In addition, the NIMI risk was positively correlated with SCC at drying off (*p* = 0.03).

Of the total cases of NIMI, CNS was the pathogen with the highest isolation frequency (*n* = 132; 46.32%), followed by *Pseudomonas* spp. (*n* = 28; 9.83%) and *Strep*. *uberis* (*n* = 21; 7.37%). According to the groups of mastitis-causing pathogens, 34% of NIMI (98/285) had isolation of major pathogens, 48% (137/285) of minor pathogens, and 18% (50/285) of other microorganisms (Table 7).

Based on the logistic regression analysis by mastitis-causing pathogens groups, SDCT protocol reduced the odds of NIMI caused by major pathogens by 1.8 times compared with MQs treated with ADCT (*p* = 0.02; Table 6). There was no effect of the drying off protocols on the occurrence of NIMI caused by minor pathogens (*p* = 0.55) and other microorganisms (*p* = 0.61).

### 3.5. Clinical Mastitis

A total of 34 quarter cases of CM were recorded during the first 60 DPP. Of these, 21 (61.8%) were identified in cows treated with ADCT protocol, while 13 (38.2%) received the SDCT treatment. The CM risk during the first 60 DPP was 2.5 cases per 100 MQ at risk for the ADCT protocol and 1.4 per 100 MQ at risk for the SDCT protocol. According to Cox regression for competing risks data, quarters assigned to the ADCT protocol were 2.79 times more likely to develop CM in the first 60 DPP than those treated with SDCT (*p* = 0.02; Table 8). However, this risk was inversely related to the parity, in which first-lactation cows had a higher CM risk until 60 DPP than multiparous cows (*p* = 0.02).

## 4. Discussion

The combined use of DCT (anhydrous cefalonium, 0.25 g) with ITS (bismuth subnitrate, 4 g) administered at the quarter level in dairy cows at drying off reduced the occurrence of postpartum NIMI and the occurrence of CM in the first 60 DPP. Similar results have been described in previous studies using different antimicrobial compounds combined with ITS and administered at drying off in cows (cloxacillin; [8,25]) and heifers before calving (amoxicillin; [16]).

In our study, the addition of ITS to the treatment protocol decreased the risk of NIMI by 1.4 times during the dry period compared to ADCT. Similarly, Golder, Hodge, and Lean [14] reported that antibiotic-treated cows were 1.9 times more likely to develop SCM than cows treated with ITS used in combination with intramammary antibiotic (cloxacillin, 600 mg). Rabiee and Lean [17] also observed reduction in NIMI occurrence (25%), with the ITS used alone or in combination with antibiotics at drying off. These results suggest that the physical barrier formed by ITS infusion at drying off reduces the risk of pathogen invasion in the MG. Teat sealants have the capacity to persist in the teat canal throughout the dry period (approximately 100 d; [26]), promoting a protection of MG against opportunistic microorganisms.

According to mastitis-causing pathogens groups, MQs treated with ITS had 1.8 times less chance of NIMI caused by major pathogens than ADCT-treated MQs. In addition, approximately 82% (80/98) of microorganisms classified as major pathogens were considered to have the environment as the main source. These results are similar to those reported by Hogan and Smith [27], who described higher rates of IMI caused by environmental pathogens during the dry period compared to lactation, especially between two weeks after drying off and the two weeks before calving [28]. The higher risk of NIMI caused by environmental pathogens may be attributed to the continuous milk production in the early days of the dry period, and subsequent engorgement of MG during this phase. These risk factors are associated with the shortening and dilatation of the teat canal, and to the malformation of the keratin plug, which facilitates the access of opportunistic microorganisms to the mammary tissue [29,30]. This condition also occurs during the accumulation of colostrum near calving [29]. Additionally, ITS-treated cows had lower occurrence of IMI caused by environmental pathogens (*Escherichia coli* and other enterobacteria) and other pathogens than cows receiving only DCT [15]. A higher volume of ITS (4 g compared to 2.6 g of bismuth subnitrate) was suggested to be associated with reduced odds of MQs presenting IMI caused by major pathogens [4], showing that the prevention of dry IMI is more related to ITS use than antimicrobials. The ITS mimics the physiological keratin plug and prevents the access of opportunistic bacteria to MG, especially those of environmental origin [15].

In our study, SDCT reduced by 2.79 times the odds of a cow acquiring CM until 60 DPP compared to ADCT treatment. Similarly to our results, Godden et al. [19] observed that MQs treated with DCT were 0.67 times more likely to have CM until 60 DPP compared to MQ treated with DCT plus ITS. Additionally, Rabiee and Lean [17] reported that DCT and ITS combination reduced the risk of CM postpartum by 29% compared with cows treated with DCT alone. Our results reinforce the ability of ITS to create a physical barrier in the teat canal and cistern against mastitis-causing pathogens access during the dry period. During that period, the MG experiences physiological and histological changes, which facilitates the access of opportunistic pathogens, especially due the incomplete or absent formation of keratin plug. Furthermore, first-lactation cows in our study had a higher CM risk until 60 DPP than multiparous cows. This result differs from that reported by Jamali et al. [31] and Pinzón-Sánchez and Ruegg [32], who observed a positive relationship between parity and CM risk. This difference in CM risk between our study and that of Jamali et al. [31] and Pinzón-Sánchez and Ruegg [32] may be attributed to the large proportion of young cows included in our study, associated with potential risk factors not controlled in our study, such as hygiene used to house cows and teat condition.

The overall bacteriological cure risk was 91% in the present study, indicating that both DCT protocols were effective in treating existing IMI. Martins et al. [4] reported similar cure risk (92%) for DCT using 0.25 g cefalonium combined with ITS. Additionally, in Italy and England, Ospina et al. [33] and Newton et al. [34] reported 90% and 94% cure risk with 0.25 g cefazoline + ITS and 600 mg cloxacillin + ITS, respectively. Despite the different antimicrobial compounds evaluated in DCT by Ospina et al. [33] and Newton et al. [34], the risks of cure were similar to that observed in our study. However, direct comparisons between studies are limited due to the different characteristics of the enrolled cows and the frequency of mastitis pathogens isolated at drying off. These characteristics associated with udder health before drying off and cow’s parity can affect the cure of IMI [5].

According to the logistic regression results, there was no effect of ITS on SCM cure risk and new SCM cases. However, when evaluated over time, both treatments were effective in reducing SCC (*p* = 0.005) after calving. Similar results were described by McParland et al. [7], who reported reduction in SCC in subsequent lactation with DCT using cefalonium. Thus, the use of antibiotics in both treatment groups in our study may explain the reduction in postpartum SCC, since treatments showed high bacteriological cure of existing IMI.

Similar to previous studies with DCT in North America [35], Europe [33], and Brazil [8], the most frequent pathogen isolated in the present study was CNS. This high frequency of CNS isolation may be associated with the ability of this group of microorganisms to colonize the teat canal during lactation or the dry period. In addition, isolation of CNS may increase near calving when the antibiotic concentration has reduced its therapeutic action in MG, allowing MG colonization [4,36]. Of the microorganisms isolated at drying off, CNS had the lowest cure percentage (83%). It is important to note that no species-level identification was performed on these isolates, and it is difficult to state whether the postpartum CNS isolates were the same that were isolated before treatment. According to Sampimon et al. [37], species-level identification of CNS based on phenotypic testing has limitations; however, molecular methods [38] and mass spectrometry techniques [39] can be used to identify CNS at the species level. However, this assessment was beyond the scope of our study.

Surprisingly, MQ infected by *Staph. aureus* presented high frequency of cure (100%) in our study, which differs from previous reports [4,40]. Shephard, Burman, and Marcun [40] reported an average of 47% cure rate for *Staph. aureus* between cows treated with DCT cefalonium-based and those treated with cloxacillin, although without difference between groups. The large discrepancy in reported cure rates between studies can be attributed to cow characteristics (e.g., mastitis records, parity, and immunological status), *Staph. aureus* strains, and treatment regimen [41]. Additionally, Barkema, Schukken, and Zadoks [41] reported that older cows are less likely to be cured from IMI caused by *Staph. aureus*, which becomes an even smaller likelihood in cows with multiple infected quarters. In our study, *Staph. aureus* isolates at drying off were identified in primiparous (31%) and multiparous (69%) cows, and 60% (12/20) of cows presented at least two infected MQ. In addition, from the total *Staph. aureus* isolates, 92% (47/51) were isolated from cows of two farms located in the same region (South of Minas Gerais state), which suggests that the *Staph. aureus* strains identified in our study were sensitive to the antimicrobial base used for treatment. A further study evaluating the antimicrobial resistance of these isolates may be suggested.

There was a difference between treatments for DIM and LSSCC, although the cows were randomly selected. Cows treated with SDCT had lower DIM and higher LSSCC compared to cows assigned to the ADCT group. These results were not expected as the cows enrolled in this study were randomly distributed among treatments. However, to control potential confounding, the effects of DIM and LSSCC on variables response evaluated were included as covariates. Although there was an initial difference between treatment groups, postpartum SCC values were numerically lower in ITS-treated cows compared with ADCT-treated cows, which corroborates with our speculation that ITS was effective in reducing postpartum NIMI.

## 5. Conclusions

In conclusion, the SDCT protocol decreased NIMI (overall and by major pathogens) and the risk of CM until 60 DPP compared to ADCT. However, the use of ITS combined with an antimicrobial had no effect on bacteriological cure, SCM cure, and new SCM cases when compared with the use of antimicrobials alone. In herds with rigorous records of individual SCC and CM occurrence during lactation, the use of ITS alone to dry off cows with no history of mastitis may be an important strategy to reduce antimicrobial use without impairing udder health in the subsequent lactation. Further research evaluating the use of ITS as an alternative method for selective dry cow therapy may be suggested.

## Figures and Tables

**Table 1:** Descriptive results of cows’ characteristics selected for the study (mean and standard deviation) and frequency of mammary quarter position according to the treatment groups evaluated.

Cows’ Characteristics	Treatment		*p*-Value
	ADCT ^a^	SDCT ^b^	
Dry period (d)	57.4 (13.1)	57.9 (13.3)	0.42
Parity (*n*)	1.98 (1.25)	1.97 (1.07)	0.84
DIM ^c^ (d)	422.9 (175.1)	395.7 (157.2)	0.0007
Quarter position (*n*, %)			
Front	420 (47.8)	458 (52.1)	0.51
Rear	420 (47.8)	458 (52.1)	
LSSCC ^d^ (Linear score)	3.80 (2.4)	4.19 (2.3)	0.001

^a^ ADCT: 0.25 g anhydrous cefalonium; ^b^ SDCT: 0.25 g anhydrous cefalonium used in combination with ITS (bismuth subnitrate 4 g); ^c^ DIM: Days in milk; ^d^ LSSCC: SCC at drying off transformed on linear score according to Schukken et al. [24].

**Table 2:** Frequency of bovine mastitis pathogens isolated from mammary quarters at drying off according to drying off protocols.

Pathogens	ADCT ^a^		SDCT ^b^		Total	
	*n*	%	*n*	%	*N*	%
Negative	657	78.21	718	78.39	1375	78.30
Gram-positive						
CNS ^c^	88	10.48	92	10.04	180	10.25
*Staph. aureus*	25	2.98	26	2.84	51	2.90
*Corynebacterium* spp.	8	0.95	22	2.40	30	1.71
*Strep. uberis*	12	1.43	9	0.98	21	1.20
*Strep. agalactiae*	13	1.55	7	0.76	20	1.14
*Strep. dysgalactiae*	6	0.71	5	0.55	11	0.63
Other *Strep.*	5	0.59	3	0.33	8	0.46
*Enterococcus* spp.	1	0.12	-	-	1	0.06
Other Gram-positive	7	0.83	8	0.87	15	0.85
Gram-negative						
*Pseudomonas* spp.	1	0.12	8	0.87	9	0.51
*Enterobacter* spp.	1	0.12	4	0.44	5	0.28
*Escherichia coli*	2	0.24	1	0.11	3	0.17
*Klebsiella* spp.	1	0.12	-	-	1	0.06
Other Gram-negative	2	0.24	1	0.11	3	0.17
Others						
Contaminated ^d^	11	1.31	12	1.31	23	1.31
Grand total	829	100.00	916	100.00	1756	100.00

^a^ ADCT: 0.25 g anhydrous cefalonium; ^b^ SDCT: 0.25 g anhydrous cefalonium used in combination with ITS (bismuth subnitrate 4 g); ^c^ CNS: Coagulase-negative staphylococci; ^d^ Contaminated > 2 pathogens isolated in the same mammary quarter sample.

**Table 3:** Frequency of bovine mastitis pathogens isolated from quarter milk samples collected at 7 ± 3 and 14 ± 3 d postpartum according to DCT protocols administered at drying off.

Pathogens	Postpartum 7 ± 3 d						Postpartum 14 ± 3 d					
	ADCT ^a^		SDCT ^b^		Total		ADCT		SDCT		Total	
	*n*	%	*n*	%	*n*	%	*n*	%	*n*	%	*N*	%
Negative	729	86.78	822	89.74	1551	88.33	714	85.00	833	90.93	1547	88.10
Gram-positive												
CNS ^c^	61	7.26	43	4.69	104	5.92	62	7.38	43	4.69	105	5.98
*Staph. aureus*	5	0.60	4	0.44	9	0.51	7	0.83	1	0.11	8	0.45
*Strep. uberis*	6	0.71	2	0.22	8	0.46	9	1.07	4	0.44	13	0.74
Other *Strep*.	2	0.24	5	0.54	7	0.40	8	0.95	4	0.44	12	0.68
*Corynebacterium* spp.	-	-	4	0.44	4	0.23	2	0.24	2	0.22	4	0.23
*Strep. dysgalactiae*	2	0.24	-	-	2	0.11	-	-	3	0.33	3	0.17
*Bacillus* spp.	-	-	-	-	-	-	2	0.24	1	0.11	3	0.17
*Enterococcus* spp.	-	-	-	-	-	-	3	0.36	-	-	3	0.17
*Strep. agalactiae*	-	-	1	0.11	1	0.06	-	-	1	0.11	1	0.06
Other Gram-positive	6	0.71	4	0.44	10	0.57	8	0.95	2	0.22	10	0.57
Gram-negative												
*Pseudomonas* spp.	11	1.31	8	0.87	19	1.08	10	1.19	3	0.33	13	0.74
*Enterobacter* spp.	1	0.12	5	0.54	6	0.34	-	-	4	0.44	4	0.23
*Escherichia coli*	3	0.36	2	0.22	5	0.28	3	0.36	1	0.11	4	0.23
*Klebsiella* spp.	1	0.12	-	-	1	0.06	1	0.12	-	-	1	0.06
Other Gram-negative	2	0.24	4	0.44	6	0.34	5	0.60	2	0.22	7	0.40
Others												
Yeast	1	0.12	2	0.22	3	0.17	1	0.12	4	0.43	5	0.28
Contaminated ^d^	10	1.19	10	1.09	20	1.14	5	0.59	8	0.87	13	0.74
Grand total	840	100.00	916	100.00	1756	100.00	840	100.00	916	100.00	1756	100.00

^a^ ADCT: 0.25 g anhydrous cefalonium; ^b^ SDCT: 0.25 g anhydrous cefalonium used in combination with ITS (bismuth subnitrate 4 g); ^c^ CNS: Coagulase-negative staphylococci; ^d^ Contaminated > 2 pathogens isolated in the same mammary quarter sample.

**Table 4:** Results of logistic regression for the effect of dry-off protocols on mammary gland health indicators (bacteriological and SCM cure and new cases of SCM).

Variable	Item	Estimate	SE ^a^	*p*-Value	OR ^b^ (95% CI ^c^)	LSM ^d^ (SEM ^e^)
Bacteriological cure ^f^						
*β* ^g^		2.21	1.04			
Treatment	ADCT ^h^	−0.84	0.46	0.07	0.43 (0.17–1.08)	0.89 (0.03)
	SDCT ^i^	Ref.				0.95 (0.02)
Quarter position	Front	−0.78	0.44	0.07	0.46 (0.20–1.09)	0.89 (0.03)
	Rear	Ref.				0.95 (0.02)
Dry period		0.02	0.02	0.27	1.02 (0.99–1.05)	
SCM cure ^j^						
*β*		2.31	0.34			
Treatment	ADCT	−0.22	0.26	0.40	0.80 (0.48–1.34)	0.85 (0.02)
	SDCT	Ref.				0.87 (0.02)
DIM ^k^		0.00	0.00	0.19	0.99 (0.99–1.00)	
New SCM case ^l^						
*β*		−1.43	0.41			
Treatment	ADCT	−0.02	0.28	0.94	0.98 (0.56–1.70)	0.13 (0.02)
	SDCT	Ref.				0.13 (0.02)
DIM		0.00	0.00	0.21	0.99 (0.99–1.00)	

^a^ SE: Standard error; ^b^ OR: Odds ratio; ^c^ CI: Confidence interval; ^d^ Least square mean; ^e^ Standard error of the mean; ^f^ Bacteriological cure: a mammary quarter was considered bacteriologically cured when the microorganism identified at drying off was not isolated in either of the two postpartum milk samples; ^g^ *β*: Regression coefficient; ^h^ ADCT: 0.25 g anhydrous cefalonium; ^i^ SDCT: 0.25 g anhydrous cefalonium used in combination with ITS (bismuth subnitrate 4 g); ^j^ Subclinical mastitis cure: considered when MQs with SCC >200,000 cells/mL before drying off had reduction to values ≤200,000 cells/mL after calving; ^k^ Days in milk; ^l^ New subclinical mastitis case: defined when MQ had SCC ≤200,000 cells/mL before drying off, with an increase to >200,000 cells/mL after calving; Variables with *p*-value > 0.30 were excluded from the final model.

**Table 5:** Risk (overall and pathogen) of bacteriological cure of mammary quarters submitted to drying off protocols.

Pathogen	Bacteriological Cure		
	ADCT ^a^	SDCT ^b^	Overall
Gram-positive	87 (143/164)	94 (160/170)	91 (303/334)
CNS ^c^	76 (67/88)	89 (82/92)	83 (149/180)
*Staph. aureus*	100 (25/25)	100 (26/26)	100 (51/51)
*Corynebacterium* spp.	100 (8/8)	100 (22/22)	100 (30/30)
*Strep. uberis*	100 (12/12)	100 (8/8)	100 (20/20)
*Strep. agalactiae*	100 (13/13)	100 (6/6)	100 (19/19)
*Strep. dysgalactiae*	100 (6/6)	100 (5/5)	100 (11/11)
Other *Strep*.	100 (5/5)	100 (3/3)	100 (8/8)
Other Gram-positive	100 (7/7)	100 (8/8)	100 (15/15)
Gram-negative	100 (7/7)	100 (14/14)	100 (21/21)
*Pseudomonas* spp.	100 (1/1)	100 (8/8)	100 (9/9)
*Enterobacter* spp.	100 (1/1)	100 (4/4)	100 (5/5)
*Escherichia coli*	100 (2/2)	100 (1/1)	100 (3/3)
*Klebsiella* spp.	100 (1/1)	-	100 (1/1)
Other Gram-negative	100 (2/2)	100 (1/1)	100 (3/3)

^a^ ADCT: 0.25 g anhydrous cefalonium; ^b^ SDCT: 0.25 g anhydrous cefalonium used in combination with ITS (bismuth subnitrate 4 g); ^c^ CNS: Coagulase-negative staphylococci.

**Table 6:** Logistic regression for the effect of drying off protocols on overall NIMI risk and by group of pathogens identified at 7 ± 3 and 14 ± 3 d postpartum.

Variable	Item	Estimate	SE ^a^	*p*-Value	OR ^b^ (95% CI ^c^)	LSM ^d^ (SEM ^e^)
NIMI ^f^						
*β* ^g^		−2.39	0.27	0.003		
Treatment	ADCT ^h^	0.38	0.14	0.007	1.46 (1.11–1.92)	0.19 (0.03)
	SDCT ^i^	Ref.				0.14 (0.02)
Quarter position	Front	0.18	0.14	0.20	1.20 (0.91–1.57)	0.17 (0.03)
	Rear	Ref.				0.15 (0.03)
Parity		0.10	0.06	0.11	1.10 (0.98–1.24)	
LSSCC ^j^		0.06	0.03	0.03	1.07 (1.00–1.13)	
Major ^k^						
*β*		−3.82	0.47	0.003		
Treatment	ADCT	0.64	0.27	0.02	1.89 (1.11–3.23)	0.06 (0.02)
	SDCT	Ref.				0.03 (0.01)
LSSCC		0.10	0.05	0.07	1.10 (0.99–1.22)	
Minor ^l^						
*β*		−2.51	0.24	0.0004		
Treatment	ADCT	−2.31	0.22	0.55	1.13 (0.76–1.69)	0.09 (0.02)
	SDCT	Ref.				0.08 (0.02)
Quarter position	Front	0.16	0.18	0.38	1.17 (0.82–1.68)	0.09 (0.02)
	Rear	Ref.				0.08 (0.02)
Others ^m^						
*β*		−4.13	0.37	0.0004		
Treatment	ADCT	0.16	0.31	0.61	1.17 (0.64–2.13)	0.03 (0.01)
	SDCT	Ref.				0.03 (0.01)
Quarter position	Front	0.27	0.29	0.36	1.30 (0.74–2.31)	0.03 (0.01)
	Rear	Ref.				0.02 (0.01)
Parity		0.17	0.12	0.14	1.19 (0.94–1.50)	

^a^ SE: Standard error; ^b^ OR: Odds ratio; ^c^ CI: Confidence interval; ^d^ Least square mean; ^e^ Standard error of the mean; ^f^ NIMI: New intramammary infection; ^g^ *β:* Regression coefficient; ^h^ ADCT: 0.25 g anhydrous cefalonium; ^i^ SDCT: 0.25 g anhydrous cefalonium used in combination with ITS (bismuth subnitrate 4 g); ^j^ LSSCC: SCC at drying transformed on linear score according to Schukken et al. [24]; ^k^ Major: major pathogens were considered *Strep*. *uberis*, *Staph*. *aureus*, Other *Strep*., *Strep*. *dysgalactiae*, *Strep*. *agalactiae*, *Escherichia coli*, *Klebsiella* spp., and *Pseudomonas* spp.; ^l^ Minor: minor pathogens were considered CNS and *Corynebacterium* spp.; ^m^ Others: other pathogens were considered other Gram-positive and Gram-negative bacteria, *Enterobacter* spp., yeast, *Enterococcus* spp., and *Bacillus* spp.

**Table 7:** Frequency of bovine mastitis pathogens isolated from NIMI cases (considering two postpartum samples) according to drying off protocols.

Pathogen	NIMI ^a^					
	ADCT ^b^ (*n* = 158)		SDCT ^c^ (*n* = 127)		Total (*n* = 285)	
	*n*	%	*n*	%	*N*	%
Major ^d^						
*Pseudomonas* spp.	18	11.39	10	7.87	28	9.83
*Strep. uberis*	15	9.50	6	4.72	21	7.37
*Staph. Aureus*	11	6.96	5	3.94	16	5.61
Other *Strep*.	8	5.06	8	6.30	16	5.61
*Escherichia coli*	5	3.16	3	2.36	8	2.81
*Strep. dysgalactiae*	2	1.27	3	2.36	5	1.75
*Strep. agalactiae*	-	-	2	1.57	2	0.70
*Klebsiella* spp.	2	1.27	-	-	2	0.70
Total	61	38.61	37	29.12	98	34.38
Minor ^e^						
CNS ^f^	71	44.94	61	48.03	132	46.32
*Corynebacterium* spp.	-	-	5	3.94	5	1.75
Total	72	44.94	66	51.97	137	48.07
Others ^g^						
Other Gram-positive	14	8.86	5	3.94	19	6.67
Other Gram-negative	5	3.16	5	3.94	10	3.51
*Enterobacter* spp.	1	0.63	8	6.30	9	3.16
Yeast	1	0.63	5	3.94	6	2.11
*Enterococcus* spp.	3	1.90	-	-	3	1.05
*Bacillus* spp.	2	1.27	1	0.79	3	1.05
Total	26	16.45	26	18.91	50	17.55

^a^ NIMI: New intramammary infection; ^b^ ADCT: 0.25 g anhydrous cefalonium; ^c^ SDCT: 0.25 g anhydrous cefalonium used in combination with ITS (bismuth subnitrate 4 g); ^d^ Major: major pathogens were considered *Strep*. *uberis*, *Staph*. *Aureus*, Other *Strep*., *Strep*. *dysgalactiae*, *Strep*. *agalactiae*, *Escherichia coli*, *Klebsiella* spp., and *Pseudomonas* spp.; ^e^ Minor: minor pathogens were considered CNS and *Corynebacterium* spp.; ^f^ CNS: Coagulase-negative staphylococci; ^g^ Others: other pathogens were considered other Gram-positive and Gram-negative bacteria, *Enterobacter* spp., yeast, *Enterococcus* spp., and *Bacillus* spp.

**Table 8:** Logistic regression for drying off protocols effect on clinical mastitis risk during the first 60 days postpartum.

Variable	Intercept	SE ^a^	HR ^b^	95% CI ^c^		*p*-Value
				Lower	Upper	
ADCT ^d^ (vs SDCT ^e^, Ref.)	1.03	0.44	2.79	1.17	6.66	0.02
Parity	−0.88	0.37	0.41	0.20	0.86	0.02
Farm	1.98	1.86	-	-	-	<0.001

^a^ SE: Standard error; ^b^ HR: Hazard ratio; ^c^ CI: Confidence interval; ^d^ ADCT: 0.25 g anhydrous cefalonium; ^e^ SDCT: 0.25 g anhydrous cefalonium used in combination with ITS (bismuth subnitrate 4 g).
